# Ethics

**Published:** 1876-10

**Authors:** 


### The Bistoury.

ELMIRA, N. Y., OCT., 1876.

The BISTOURY is published Quarterly, upon the 1st of April, July, October and January, at Fifty-five Cents a year, in advance.

### ETHICS.

An editorial article in a recent number of *New Remedies*, discusses a meeting held in New York, where several prominent medical gentlemen, some of whom were professors of the several medical schools of the city, took strong grounds, in speeches made upon the occasion, in favor of consulting with homeopathic physicians. It seems, from the article referred to, that the sentiment was heartily endorsed by most of the medical gentlemen present; and, although we do not know the ground upon which they based their conclusions, yet we are prepared to admire and endorse their wisdom.

Heretofore, the code of ethics has been so interpreted, as to prohibit the consultation of any "regular" practitioner with a homeopathic physician, so causing our homeopathic brethren to appear before the public as martyrs in an eminently just cause. It cannot be disputed that the ranks of the homeopathic fraternity contain many well educated gentlemen, who by reason of intellectual endowments and social standing, are entitled to our consideration and respect. They are employed by our most respectable and best informed citizens, and in the cities, generally monopolize what is termed the "cream of the practice" among the wealthy and best paying classes. That they are successful, too, in the management of the sick, cannot be disputed, therefore, intelligence, education, social standing and success, *should be* sufficient endorsement to recommend them to our consideration.

If their system is based upon false theory, no better place exists to convince them of it than in the regular medical societies. Admit them, therefore, to your deliberations, and by fair and open discussion, convince them of their folly, if you can, or listen to their theories and arguments, and believe just so much as cannot be contradicted. We are sure that both parties can be improved, one by the other, in mutual controversy or discussion of this character. If we are right, and we firmly believe our theroy of practice to be the true one, we should not fear to have it assailed by any body of men. If it is false or faulty, intelligent discussion can have no harmful influence upon it, but contrawise, improve and enlarge the practice of medicine, to the mutual benefit of the honest physician and the people.

